# Microscopic evaluation of spin and orbital moment in ferromagnetic resonance

**DOI:** 10.1038/s41598-024-66139-1

**Published:** 2024-07-05

**Authors:** Yuta Ishii, Yuichi Yamasaki, Yusuke Kozuka, Jana Lustikova, Yoichi Nii, Yoshinori Onose, Yuichi Yokoyama, Masaichiro Mizumaki, Jun-ichi Adachi, Hironori Nakao, Taka-hisa Arima, Yusuke Wakabayashi

**Affiliations:** 1 Department of Physics, Tohoku University, Sendai, 980-8578 Japan; 2 PRESTO, Japan Science and Technology Agency (JST), Kawaguchi, Japan; 3 National Institute for Materials Science (NIMS), Tsukuba, 305-0047 Japan; 4 RIKEN Center for Emergent Matter Science (CEMS), Wako, 351-0198 Japan; 5 Center for Science and Innovation in Spintronics, Tohoku University, Sendai, 980-8577 Japan; 6 Institute for Materials Research, Tohoku University, Sendai, 980-8577 Japan; 7 Japan Synchrotron Radiation Research Institute (JASRI/SPring-8), Sayo, 679-5198 Japan; 8 Faculty of Science, Course for Physical Sciences, Kumamoto University, Kumamoto, 860-0862 Japan; 9 Photon Factory, Institute of Materials Structure Science, High Energy Accelerator Research Organization, Tsukuba, 305-0801 Japan

**Keywords:** Ferromagnetism, Magnetic properties and materials, Spintronics, Characterization and analytical techniques

## Abstract

Time-resolved X-ray magnetic circular dichroism under the effects of ferromagnetic resonance (FMR), known as X-ray ferromagnetic resonance (XFMR) measurements, enables direct detection of precession dynamics of magnetic moment. Here we demonstrated XFMR measurements and Bayesian analyses as a quantitative probe for the precession of spin and orbital magnetic moments under the FMR effect. Magnetization precessions in two different Pt/Ni-Fe thin film samples were directly detected. Furthermore, the ratio of dynamical spin and orbital magnetic moments was evaluated quantitatively by Bayesian analyses for XFMR energy spectra around the Ni $L_{2,3}$ absorption edges. Our study paves the way for a microscopic investigation of the contribution of the orbital magnetic moment to magnetization dynamics.

## Introduction

The magnetization dynamics related to orbital anglar momentum (OAM) has recently gained significant attention in various research fields. It is expected to play a pivotal role in the advancement of spintronics and orbitronics devices. Although OAM is frequently considered to be quenched in solids due to the strong crystalline field^1^, several mechanisms have been proposed to revive OAM, including spin-orbit (SO) coupling^2,3^, and emergent magnetic fields generated by non-collinear spin textures^4–6^. OAM can also persist in nonequilibrium states under particular conditions, which give rise to distinctive OAM dynamical phenomena such as the orbital magnetic moment of magnon and orbital current that carries the OAM^7–14^.

**Figure 1:**
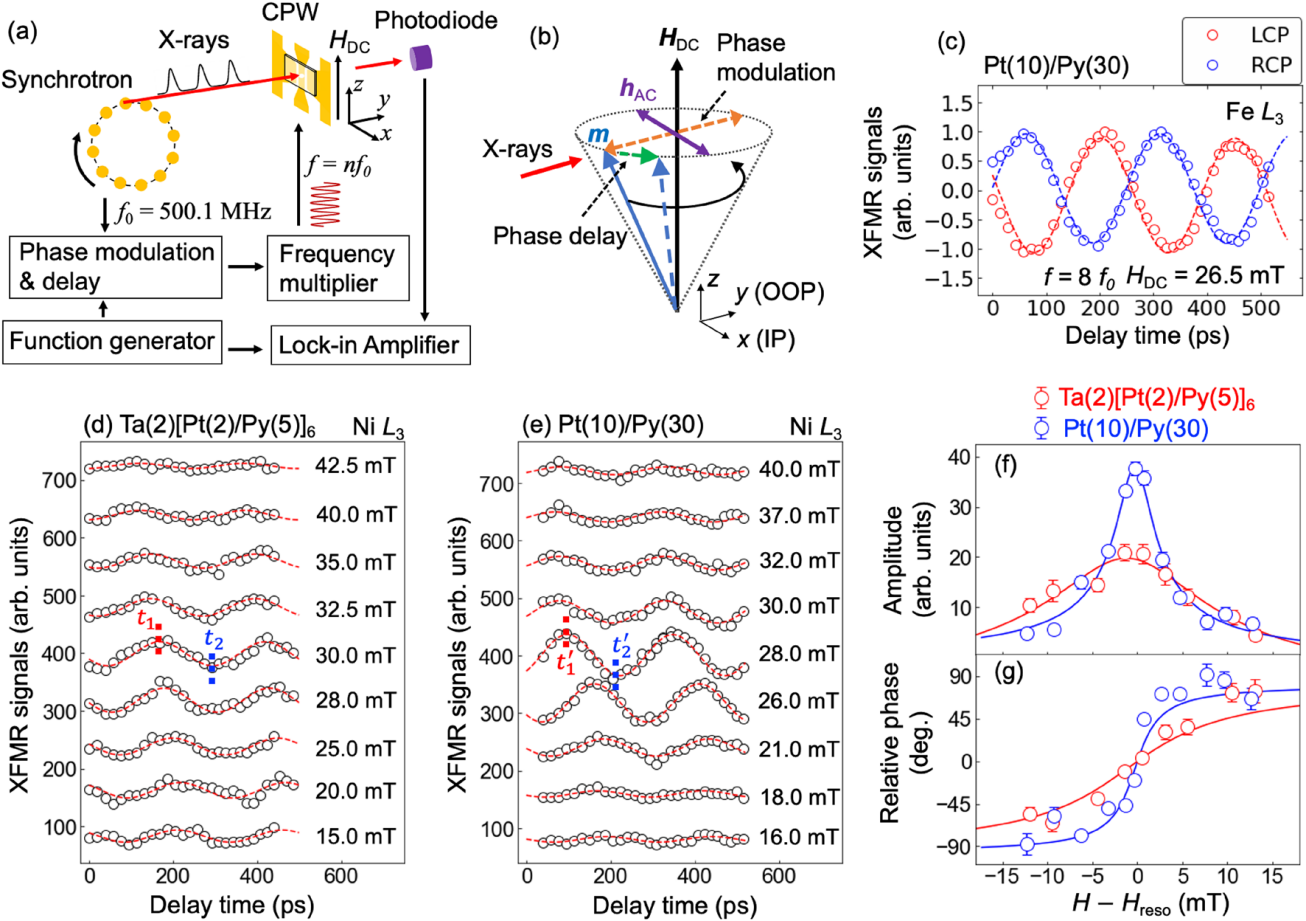
(**a**) Experimental configuration of XFMR measurement. The RF master oscillating signals with a frequency of $f_0 = 500.1 \hbox {MHz}$ are employed as the RF field for FMR effect. The RF signals are delivered to the sample via a coplanar waveguide (CPW) after delaying the phase and multiplying the frequency. The phase of the RF field is also modulated by $\pi$ with a frequency of 1.0333 kHz by using a square-wave signal generated from a function generator. The square-wave signal and the detected XMCD signals were fed into a Lock-in Amplifier. A bias magnetic field is applied perpendicular to the direction of X-rays and AC magnetic field of the RF field. (**b**) Schematic representation of magnetization precession under FMR effect with the modulated RF field. $\textbf{m}$ represents a magnetic moment, and IP and OOP present in-plane and out-of-plane directions to a sample surface, respectively. (**c**) Phase delay scans of XFMR signals for Pt(10)/Py(30) sample at the Fe $L_3$ edge obtained by using left and right circular polarized (LCP and RCP) X-rays. (**d**,**e**) Phase delay scans for (**d**) Ta(2)[Pt(2)/Py(5)]$_{6}$ and (**e**) Pt(10)/Py(30) samples at various bias magnetic fields across the ferromagnetic resonance field. RF field with a frequency $f = 4.0008$ GHz was applied for the samples. Dashed lines indicate $t_1 = 160\,\hbox {ps}$ and $t_2 = 290\,\hbox {ps}$ for Ta(2)[Pt(2)/Py(5)]$_{6}$, and $t_1^{\prime } = 90\,\hbox {ps}$ and $t_2^{\prime } = 210\,\hbox {ps}$ for Pt(10)/Py(30). (**f**,**g**) Bias magnetic field dependence of (**f**) the amplitude and (**g**) the relative phase of the magnetization precessions. Red and blue lines represent the fitting results.

One of the candidate probes for the dynamics of orbital magnetic moment is X-ray magnetic circular dichroism (XMCD). This technique enables to detect magnetic moments along the incident X-ray direction by measuring the difference in absorption of left- and right-circularly polarized X-rays, and to separate the contributions of spin and orbital magnetic moments (referred to as $m_S$ and $m_L$, respectively) using the magneto-optical sum rules for XMCD energy spectrum^15–17^. Furthermore recent time-resolved (Tr-) XMCD measurement has reported direct detection of the transient dynamics of $m_S$ and $m_L$ induced by optical laser pulses^18^.

Tr-XMCD has also been applied to the stroboscopic real-time detection of magnetization dynamics induced by the ferromagnetic resonance (FMR) effect, where the radio-frequency (RF) field excites magnetization precessions. This technique, known as X-ray ferromagnetic resonance (XFMR), has been utilized to directly detect the precession of magnetic moments for various magnetic samples^19–41^, and to visualize magnon propagation using scanning transmission X-ray microscopy and holographic techniques^42–56^. However, reports elucidating the quantitative separation and the detection of the precession of $m_S$ and $m_L$ are limited. This is partially due to the relatively weak signal intensities obtained from XFMR measurements, which is typically a factor of $10^{-2}$ lower than those obtained from XMCD measurements because of the small precession angle exited by the FMR effect. While a few XFMR measurements have been performed at the Fe *K* absorption edge to detect $m_L$ dynamics of Fe ion^57^, these experiments mainly provided information on the magnetic moment of the 4*p* electron, rather than the 3*d* electron. Therefore, it is crucial to establish the XFMR technique as a quantitative probe capable of directly evaluating $m_L$ dynamics of the 3*d* or 4*f* electrons in magnetic materials. This would lead to advancements in our understanding of the magnetic phenomena related to OAM.

In the present study, we demonstrated XFMR measurements in conjunction with Bayesian analysis to quantitatively detect $m_S$ and $m_L$ in the magnetization precession of $\hbox {Pt/Ni}_{0.8}\hbox {Fe}_{0.2}$ (Py) thin film samples. XFMR measurements were performed at the Fe and Ni $L_{2,3}$ absorption edges, which enables to directly detect magnetization dynamics of the Fe and Ni 3*d* electrons. Notably, Bayesian analysis has recently been successfully utilized in XMCD spectral analysis, which enables the determination of $m_S$ and $m_L$ values, and the uncertainties derived from the standard deviations of the posterior probability distributions^58^. Accordingly, we employed a Bayesian analysis for XFMR spectra and evaluated $m_L$ precession under the FMR effect. Furthermore, Pt or other 5*d* heavy metal elements are frequently employed as spin-sink or to provide spin-Hall effects. We synthesized two different types of thin films with Pt to evaluate the effects of 5*d* ions on the $m_L$ dynamics under the FMR effects.

**Figure 2:**
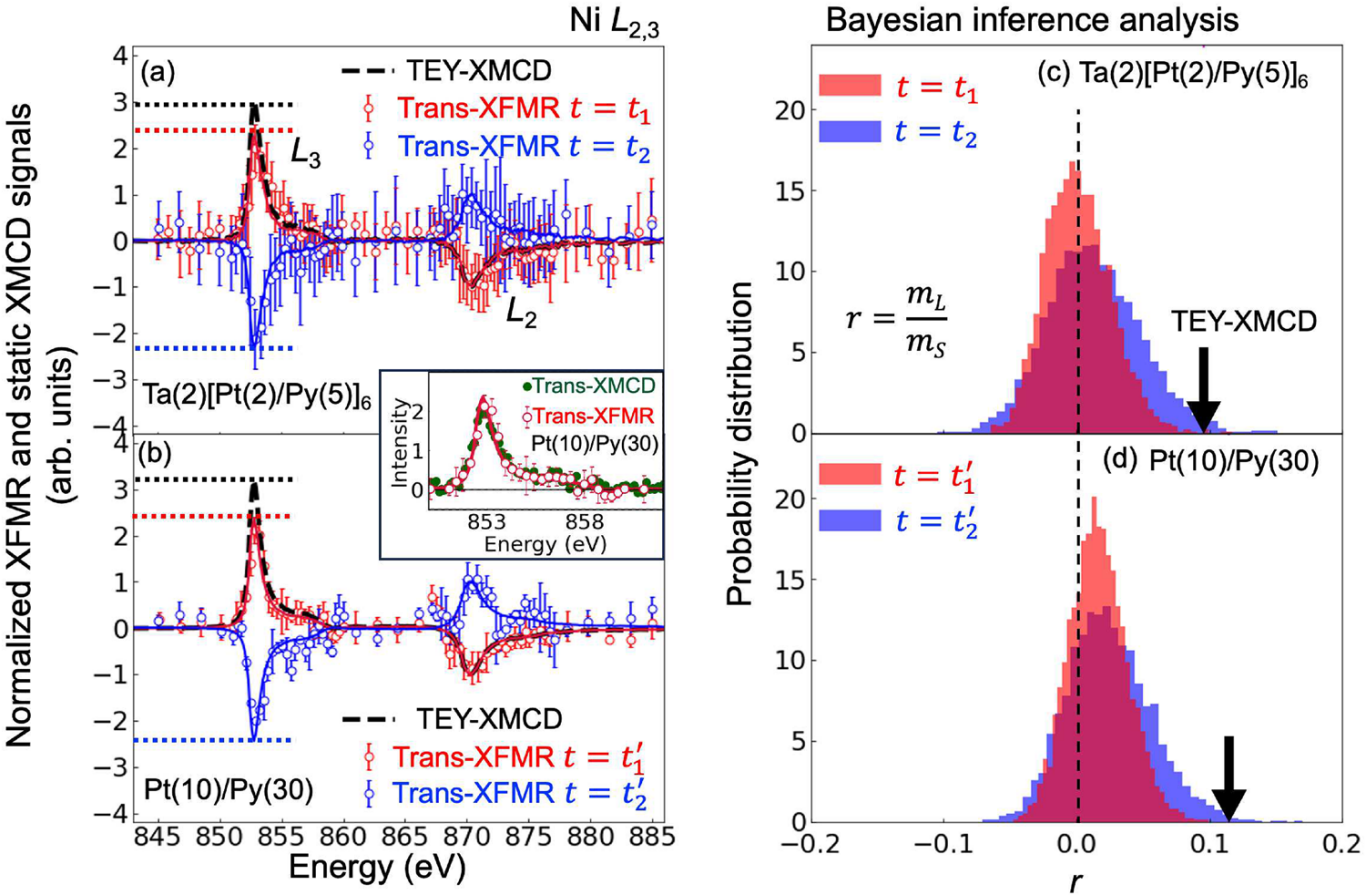
(**a**,**b**) Energy spectra of XFMR and TEY-XMCD signals around the Ni $L_{2,3}$ edges for (**a**) Ta(2)[Pt(2)/Py(5)]$_{6}$ and (**b**) Pt(10)/Py(30). The XFMR data were obtained at phase delay times of $t = t_1$ and $t = t_2$ for Ta(2)[Pt(2)/Py(5)]$_{6}$, and $t = t_1^{\prime }$ and $t = t_2^{\prime }$ for Pt(10)/Py(30) (see Fig. 1d,e). Red and blue lines represent the estimated XFMR spectra obtained by Bayesian inference analysis. The TEY-XMCD spectra are represented by dashed lines. All spectra were normalized to the intensity at the $L_2$ edge for comparison. Dotted lines indicate values of the intensities at the $L_3$ edge of the TEY-XMCD signals and the estimated XFMR spectra. Inset in the figure (**b**) shows Trans-XMCD and XFMR spectra around the $L_3$ edge for Pt(10)/Py(30). (**c**,**d**) Posterior probability distribution of the ratio of $m_L$ to $m_S$ ($r = m_L / m_S$) obtained by Bayesian inference analysis. The values of *r* obtained by the TEY-XMCD spectra were also represented as black arrows.

## Results and discussion

### XFMR measurement

XFMR measurements were performed for two different Pt/Py thin-film samples, consisting of a Ta(2)[Pt(2)/Py(5)]$_{6}$ multilayered composition and a Pt(10)/Py(30) bilayered composition (numbers in parentheses indicate film thickness in nanometers) deposited on SiN membranes. The experimental configuration is depicted in Fig. 1a (see details in the “Methods” section). The magnetization precession was excited by applying the RF magnetic field $\textbf{h}_\text{AC}$, which is the higher harmonics of the synchrotron master oscillating signal ($f_0 = 500.1\,\hbox {MHz}$). By incrementally delaying the phase of the RF field with respect to the timing of X-ray injection, the progression of magnetization precession can be monitored. Furthermore, the phase of the RF field was modulated by $\pi$ by using square-wave signals. This square-wave signal, along with the transmitted XMCD signals detected by a photodiode, were fed into a lock-in amplifier (LIA) to enhance the signal-to-noise ratio. The output signal from the LIA (referred to as XFMR signal) corresponded to the difference in the X-ray absorption between opposite sides of the cone of magnetization precession, as demonstrated in Fig. 1b.

Typical results of phase delay scans are shown in Fig. 1c. These data were obtained for the Pt(10)/Py(30) sample during XFMR measurements at the Fe $L_3$ edge (incident X-ray energy $E = 708.2\,\hbox {eV}$). The sample was subjected to an RF field with a frequency of $f = 8f_0 = 4.0008\,\hbox {GHz}$ and a bias magnetic field $H_\text{DC} = 26.5$ mT. The data exhibited well-defined oscillations and a periodicity of approximately 250 ps, corresponding to the frequency of the applied RF field. It was also confirmed that the phase of the XMFR signal is shifted by $\pi$ between left and right circularly polarized (LCP and RCP) X-rays. These results clearly indicate successful detection of magnetization precession induced by the FMR effect.

Figure 1d,e present phase delay scans for the Ta(2)[Pt(2)/Py(5)]$_{6}$ and Pt(10)/Py(30) samples at the Ni $L_{3}$ edge ($E = 853.4$ eV) under different $H_\text{DC}$ across the ferromagnetic resonance field $H_\text{reso}$. The RF field with a frequency of $f = 4.0008$ GHz was applied to the samples. Oscillating signals were clearly detected for both samples. By fitting the experimental results, we obtained the magnetic field dependence of the amplitude and phase of the precession, as shown in Fig. 1f,g. The difference in the phase between two samples is probably due to some artifacts, such as a difference in the length of microwave cables used in these measurements. Hence we defined the phase of the data on $H_\text{reso}$ as zero for both samples, and the relative phase values are plotted in Fig. 1g. The amplitudes reach the maximum values at $H_\text{reso}$, and the phase of the precession with respect to the RF field phase exhibit drastic changes around $H_\text{reso}$, in agreement with results reported in previous experiments for total angular magnetic moment $m_J$^25,26,28–31^.

Here we define that $H_\text{DC}$ is applied along the *z* direction and the incident X-ray beam is along the *y* direction, as shown in Fig. 1b. In this geometry, the incident X-ray detects *y*-components of the precession, which is expressed by the following equation (see details in Supplemental Information S1) :

1 $$\begin{aligned} m_y = A\sin (\omega t + \theta ), \end{aligned}$$

where $\omega = 2\pi f$ denotes the angular frequency of the RF field, $A \propto \sqrt{(\chi ^{yx}_1)^2 + (\chi ^{yx}_2)^2 }$, and $\theta = \arctan (- \chi ^{yx}_2/ \chi ^{yx}_1)$. $\chi ^{yx}_1$ and $\chi ^{yx}_2$ are the real and imaginary parts of the off-diagonal components of the in-plane magnetic susceptibility, respectively, which are given by Eqs. (S1.4) and (S1.5) in Supplemental information. Amplitudes and relative phases of the experimental data were analyzed via curve fitting using Eq. (1). As depicted in Fig. 1f,g, the analysis results accurately reproduced the experimental data, and provided the values of deduced Gilbert damping factors of $\alpha = 0.06 (2)$ for the Ta(2)[Pt(2)/Py(5)]$_{6}$ and $\alpha = 0.016 (1)$ for the Pt(10)/Py(30) samples, respectively. The pronounced damping factor observed for the Ta(2)[Pt(2)/Py(5)]$_{6}$ can be attributed to the interfacial effects between Pt and Py layers, including spin-flip^59,60^ and spin-pumping^61^ processes.

### XFMR spectra and Bayesian analysis

To reveal the contribution of $m_L$ to the magnetization precession, energy spectra of XFMR signals around the Ni $L_{2,3}$ edges were measured at the resonant magnetic field (as shown in Fig. 2a,b). These spectra were acquired at phase delay times of $t_1 = 160\,\hbox {ps}$ and $t_2 = 290\,\hbox {ps}$ for the Ta(2)[Pt(2)/Py(5)]$_{6}$, and, $t^{\prime }_1 = 90\,\hbox {ps}$ and $t^{\prime }_2 = 210\,\hbox {ps}$ for the Pt(10)/Py(30), which are indicated in Fig. 1d,e. We observed well-defined peaks around both $L_{2,3}$ edges, akin to a typical XMCD spectrum. Static XMCD spectra were obtained using the total-electron-yield (TEY) method (TEY-XMCD), where an external magnetic field was applied perpendicular to the sample surface, which are represented by the dashed black lines in Fig. 2a,b. Additionally, static XMCD were also acquired by transmission method (Trans-XMCD) for the Pt(10)/Py(30), shown in the inset of Fig. 2b. All spectra were normalized to the intensities at $L_2$ edge to compare the integral intensities around $L_2$ and $L_3$ edges. The XFMR spectra are almost the same as the Trans-XMCD, while the intensities at $L_3$ edge of those spectra are smaller than those of the TEY-XMCD, as indicated by dashed lines. This suggests that the ratio values of $m_L$ to $m_S$ deduced from the XFMR and Trans-XMCD spectra are different from those of the TEY-XMCD spectra.

The XFMR spectra can be analyzed by the magnetic-optical sum rule equations^15–17^, because XFMR measurement detects the projected components of XMCD signals along the X-ray direction, which reflects the magnetic state of the momentary state at each delay time (see also Supplemental Information S2). The equations are expressed as follows :

2 $$\begin{aligned} m_S\left( 1 + \frac{7}{2}{T_z}\right)= & {} -n_h \frac{6p-4q}{s}, \end{aligned}$$

3 $$\begin{aligned} m_L= & {} -n_h \frac{4q}{3s}, \end{aligned}$$

where $T_z$ represents magnetic dipole term, *s* is the integral of XAS spectrum over the $L_{2,3}$ edges, and $n_h$ represents the number of holes in 3*d* band. *p* and *q* represent the integrals of the spectra over the $L_3$ and $L_{2,3}$ edges, respectively. We ignore $T_z$ term in Eq. (2) because the samples were polycrystalline and the contributions of the shapes of the thin film samples to this term are negligible. Thus, the ratio of $m_L$ to $m_S$ can be written as

4 $$\begin{aligned} r:= \frac{m_L}{m_S} = \frac{2}{3} \cdot \frac{q}{3p-2q}. \end{aligned}$$

As noted above, intensities at the $L_3$ edge of the TEY-XMCD spectra exceed those of the XFMR and the Trans-XMCD spectra, indicating large *q* values for the TEY-XMCD data.

For further quantitative analysis, Bayesian analysis was employed to estimate the values of *r* and the uncertainties for the XFMR spectra (see details in Supplemental Information S4).

In the analysis, we utilized the static TEY-XMCD spectra as fitting functions to extract integral intensities of the XFMR spectra. We define $\varvec{\theta } = \{C_1,C_2\}$ as a parameter set for Bayesian analysis, where $C_1$ and $C_2$ represent the constant coefficients of multiplication for the spectra around the Ni $L_3$ and $L_2$ edges. The posterior probability distribution $P_{\varvec{\theta }}$ was calculated by sampling the parameter $\varvec{\theta }$ using Replica exchange Monte Carlo method. Subsequently, the posterior probability distribution of *r* (referred to as $P_r$) was obtained from $P_{\varvec{\theta }}$ by utilizing of Eq. (4). Fig. 2c,d show $P_r$ for the XFMR and TEY-XMCD data. The most probable values of *r* were obtained as $r = 0.00 (2)$ and $r = 0.01 (3)$ for $t = t_1$ and $t = t_2$ for Ta(2)[Pt(2)/Py(5)]$_{6}$, and $r = 0.01 (2)$ and $r = 0.02 (3)$ for $t = t_1^{\prime }$ and $t = t_2^{\prime }$ for Pt(10)/Py(30) for the XFMR spectra, where the values of *r* and the uncertainties were derived from the maximum values and standard deviations of $P_r$. While the maximum values of $P_r$ were obtained around $r = 0$ within the uncertainties for both samples, it is worth noting that the mean values of the $P_r$ for Pt(10)/Py(30) are slightly deviated from $r = 0$ as shown in Fig. 2d. Bayesian analyses provided the estimated XFMR spectra, indicated by red and blue solid lines in Fig. 2a,b, which accurately reproduced the experimental data for both samples. This confirms the appropriateness of utilizing TEY-XMCD spectra as fitting functions. In contrast, values of *r* acquired from the TEY-XMCD spectra represented by the black arrows in Fig. 2c,d, are $r = 0.10$ for Ta(2)[Pt(2)/Py(5)]$_{6}$ and $r = 0.11$ for Pt(10)/Py(30). The results indicate disappearance of the $m_L$ components in the XFMR and Trans-XMCD data even though the TEY-XMCD measurement detects the finite $m_L$.

These results can be explained by the different values of $m_L$ between the surface and bulk of the samples. $m_L$ is known to be enhanced at the sample surface due to lowering of the symmetry^62,63^. TEY method detects the magnetic state at sub-nm depth from the sample surface, whereas transmission measurement probes the bulk of the sample. Thus the $m_L$ components were detected by only the TEY-XMCD measurements.

Furthermore the estimated *r* for the Pt(10)/Py(30) are slightly deviated from $r = 0$, whereas *r* completely disappears for the multilayered sample of Ta(2)[Pt(2)/Py(5)]$_{6}$. This difference might be attributed to the perpendicular anisotropy of $m_L$. $m_L$ is preferably oriented normal to the sample surface in a multilayered system comprising 3*d* magnetic metal and 5*d* non-magnetic metal with strong SO coupling. 3*d*-5*d* hybridization at the interface enhances the perpendicular $m_L$^64–66^. This indicates an inclination of the precession axis of $m_L$ from the sample surface plane, resulting in a reduction in the perpendicular dynamical component of $m_L$ precession in Ta(2)[Pt(2)/Py(5)]$_{6}$.

It is noteworthy that our present measurements provided microscopic evaluation of $m_L$ in the FMR precession. Moreover, applying the present technique to imaging measurements leads to the direct space-resolved detection of the dynamics of $m_L$, such as magnons of $m_L$^7^.

## Conclusion

We performed XFMR measurements and Bayesian analysis on two different structured Pt/Py thin-film samples. The present investigation provides a microscopic and quantitative measurement techniques of orbital magnetic moment in magnetization dynamics.

## Methods

### Sample preparation

The polycrystalline thin film samples were prepared by DC magnetron sputtering under an atmosphere of 5 mTorr of Ar at ambient temperature. The power supplied to the sputtering source was 30 W for the Pt, 40 W for Ta, and 50 W for Py ($\hbox {Ni}_{0.8}\hbox {Fe}_{0.2}$). The base pressure of the chamber was $1 \times 10^{-8}$ Torr.

### XFMR and XMCD experiment

XFMR measurements were performed at BL-16A, the Photon Factory at KEK, Japan. The Photon Factory provided pulsed X-rays with a width of approximately 50 ps and the repetition rate was set to $f_0 = 500.1\,\hbox {MHz}$. For the time-resolved measurements, the synchrotron master oscillating signals were used. The phase of this signal was delayed and modulated by $\pi$ using a phase shifter controlled by square-wave signals with a frequency of 1.0333 kHz from a function generator. Subsequently, the frequency of this signal was multiplied by an integer using a multiplier, and a bandpass filter was employed to remove all harmonics except the desired ones. The RF field, with an amplitude amplified to 4 dBm by an amplifier, was then introduced into the sample via a coplanar waveguide (CPW) with a 200-μm-diameter through-hole at its center, allowing X-rays to penetrate the CPW. Transmitted X-rays were detected by a photodiode set downstream of the sample. To perform the stroboscopic XMCD measurements, this RF field must be the higher harmonics of the synchrotron master oscillating signals. As shown in Fig. 1b, for all XFMR measurements, the bias magnetic field $H_\text{DC}$ and the RF magnetic field $\textbf{h}_\text{AC}$, which was perpendicular to each other, were applied along to the in-plane to the sample surface, and X-rays were coming to the direction of out-of-plane to the sample surface.

Static XMCD spectra were acquired in the total-electron-yield (TEY) mode. The XMCD data were obtained by reversing the right and left circular polarizations of the X-rays at a frequency of 10 Hz for each photon energy^67^.

All the measurements were conducted at room temperature.

### Supplementary Information

Supplementary Information.

## Data Availability

The data supporting the findings of this study are available from the corresponding author upon reasonable request.
