# Supplementary material for: Microscopic evaluation of spin and orbital moment in ferromagnetic resonance
Source: Sci Rep. 2024 Jul 5;14:15504. doi: 10.1038/s41598-024-66139-1 (PMC11226459; doi:10.1038/s41598-024-66139-1)
Supplement: Supplementary file 1 — Supplementary Information. [file 41598_2024_66139_MOESM1_ESM.pdf]

Supplemental information for Microscopic evaluation of orbital moment in ferromagnetic resonance

Yuta Ishii[1,2,*], Yuichi Yamasaki[3,4], Yusuke Kozuka[3], Jana Lustikova[5], Yoichi Nii[6], Yoshinori Onose[6], Yuichi Yokoyama[7], Masaichiro Mizumaki[7], Junichi Adachi[8], Hironori Nakao[8], Taka-hisa Arima[4], and Yusuke Wakabayashi[1]

[1]Department of Physics, Tohoku University, Sendai 980-8578, Japan
[2]PRESTO, Japan Science and Technology Agency (JST)
[3]National Institute for Materials Science (NIMS), Tsukuba, 305-0047, Japan
[4]RIKEN Center for Emergent Matter Science (CEMS), Wako 351-0198, Japan
[5]Center for Science and Innovation in Spintronics, Tohoku University, Sendai 980-8577, Japan
[6]Institute for Materials Research, Tohoku University, Sendai 980-8577, Japan
[7]Japan Synchrotron Radiation Research Institute (JASRI/SPring-8), Sayo 679-5198, Japan
[8]Photon Factory, Institute of Materials Structure Science, High Energy Accelerator Research Organization, Tsukuba 305-0801, Japan
*yuta.ishii.c2@tohoku.ac.jp

S1. Magnetic precession in FMR detected by XFMR

Magnetization precession in the ferromagnetic resonance is expressed by the Landau-Lifshitz-Gilbert (LLG) equation, which is given by,

$$\frac{\partial \mathbf{M}}{\partial t} = -\mu_0\gamma\mathbf{M}\times\mathbf{H}_{\mathrm{eff}} + \frac{\alpha}{M}\left(\mathbf{M}\times\frac{\partial \mathbf{M}}{\partial t}\right), \tag{S1.1}$$

where $\mathbf{M}$ represents a magnetic moment vector, M is a norm of $\mathbf{M}$, and μ_0, γ, and α are magnetic permeability of vacuum, electron gyromagnetic ratio, and Gilbert damping factor, respectively. $\mathbf{H}_{\mathrm{eff}}$ is effective magnetic field including a bias magnetic field H_{DC}, RF magnetic field $\mathbf{h}_{\mathrm{AC}}$, and demagnetization field along the surface normal of the sample. Here we defined that H_{DC} is applied along the z direction and the incident X-ray beam is along the y direction as shown in Fig.1 (b) in the main text. $\mathbf{h}_{\mathrm{AC}}$ is produced around the signal line of the coplanar waveguide, so that it can be regarded that $\mathbf{h}_{\mathrm{AC}}$ is approximately along the x direction for the sample. In this geometry, it can be assumed that magnetization precession occurs in the xy plane; that is to say, magnetic moment vector can be defined as $\mathbf{M} = (m_x, m_y, M)$ where $M >> m_x, m_y$. $\mathbf{H}_{\mathrm{eff}}$ is expressed as,

$$\mathbf{H}_{\mathrm{eff}} = \begin{pmatrix} h^x \\ -m_y \\ H_{\mathrm{DC}} \end{pmatrix}, \tag{S1.2}$$

where $h^x = \tilde{h}^x \exp(i\omega t)$, $\tilde{h}^x$ is the amplitude of $\mathbf{h}_{\mathrm{AC}}$, $\omega = 2\pi f$ is the angular frequency of the RF field. Demagnetization field is represented by y component of $\mathbf{H}_{\mathrm{eff}}$.

Because XMCD measurement provides the information of a component of a magnetic moment along the direction of the incident X-rays, XFMR measurement detects y-components of the $\mathbf{M}$, which is expressed by using the off-diagonal component of the in-plane magnetic susceptibility χ^{yx} as below,

$$m_y = \chi^{yx} h^x. \tag{S1.3}$$

According to Eq. (S1.1), real and imaginary parts of χ^{yx}, χ_1^{yx} and χ_2^{yx}, can be expressed as below,

$$\chi_1^{yx} = \frac{-\alpha\omega_M(2\omega_H+\omega_{\mathrm{M}})\omega^2}{[(1+\alpha^2)\omega^2-\omega_0^2]^2+\alpha^2\omega^2(2\omega_H+\omega_{\mathrm{M}})^2}, \tag{S1.4}$$

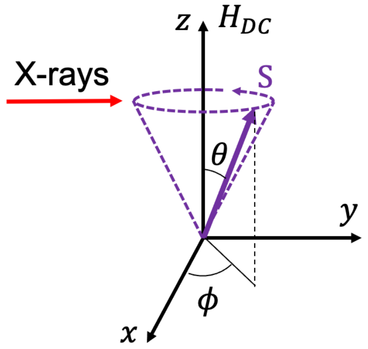

Fig. S1: Experimental geometry of our XFMR measurements.

and

$$\chi_2^{yx} = \frac{\omega\omega_M[(1+\alpha^2)\omega^2 - \omega_0^2]}{[(1+\alpha^2)\omega^2 - \omega_0^2]^2 + \alpha^2\omega^2(2\omega_H + \omega_{\rm M})^2} \tag{S1.5}$$

where $\omega_0 = \mu_0\gamma\sqrt{H_{\rm DC}(H_{\rm DC}+M)}$, $\omega_H = \mu_0\gamma H_{\rm DC}$, and $\omega_M = \mu_0\gamma M$. By using these components, m_y is represented by the below equation,

$$m_y = A\sin(\omega t + \theta), \tag{S1.6}$$

where

$$A = \tilde{h}^x\sqrt{(\chi_1^{yx})^2 + (\chi_2^{yx})^2} \tag{S1.7}$$

and

$$\theta = \arctan(-\chi_2^{yx}/\chi_1^{yx}). \tag{S1.8}$$

S2. Application of XMCD equations for the XFMR measurements

Here we describe an application of XMCD equations for the present XFMR experiments. In the present experiments, microwaves excite magnetization precession continuously, and X-rays with a pulse width of 50 ps detect the moments at each delay time. This time scale is much longer than those of the electronic repopulation and thermalization ($\delta t < 1$ ps), which have been investigated by ab-initio calculations for pulse laser pump and x-ray probe experiments [1], as well as inter-band ($2p$-$3d$) electronic transition in XMCD process ($\delta t < 1$ fs). Thus, our XFMR measurement detects the magnetic moments at an almost steady state at each delay time.

XMCD signal ($\Delta\gamma$), which is a difference between X-ray attenuations acquired with left and right circular polarized X-rays, is expressed by the below equations [2];

$$\Delta\gamma = C\Im(F_{-1}^1 - F_{+1}^1), \tag{S2.1}$$

$$F_{\pm 1}^1 = \sum_b p(b)\frac{|Q_\pm|^2}{E_b - E_a + E_p + i\Gamma/2}, \tag{S2.2}$$

$$Q_\pm = \langle a|\hat{R}_{\pm 1}|b\rangle\,. \tag{S2.3}$$

We assumed that $H_{\rm DC}$ and incident X-ray beams are parallel to z- and y-axes, respectively, as shown in Fig. S1. In Eqs. (S2.1)∼(S2.3), $|a\rangle$ and $|b\rangle$ represent the initial and the intermediate states with the energies E_a and E_b, respectively. $\hat{R}_{\pm 1}$ is defined as $\hat{R}_{\pm 1} = \mp 1/\sqrt{2}(\hat{R}_z \pm i\hat{R}_x)$ (and $\hat{R}_0 = \hat{R}_y$) with Cartesian components of the position operator $\hat{R}_{x,y,z}$. $p(b)$, E_p, Γ, and C refer to probability of the final state, the energy of the photon, the decay width, and coefficient, respectively.

To simplify, we now consider a one-electron process. The spin state of the final state can be given by

$$|S, m_s\rangle_b = \cos\frac{\theta}{2}\left|\frac{1}{2},\uparrow\right\rangle_z + {\rm e}^{i\phi}\sin\frac{\theta}{2}\left|\frac{1}{2},\downarrow\right\rangle_z \tag{S2.4}$$

$$= \frac{1}{\sqrt{2}}\left[\left(\cos\frac{\theta}{2} - ie^{i\phi}\sin\frac{\theta}{2}\right)\left|\frac{1}{2},\uparrow\right\rangle_y + \left(\cos\frac{\theta}{2} + ie^{i\phi}\sin\frac{\theta}{2}\right)\left|\frac{1}{2},\downarrow\right\rangle_y\right] \tag{S2.5}$$

where θ and ϕ are polar coordinates, shown in Fig. S1. $|\frac{1}{2}, \uparrow\rangle_{z(y)}$ is a spin state of an electron with bases when the z (y) direction is chosen as a quantization axis. Using this notation, $|b\rangle$ can be expressed by

$$\begin{aligned} |b\rangle &= \sum C_{L,m_l,S,m_s} |L, m_l\rangle_b |S, m_s\rangle_b \\ &= \sum C_{L,m_l,S,m_s} |L, m_l\rangle_b \\ &\times \left(\frac{1}{\sqrt{2}} \left[\left(\cos\frac{\theta}{2} - ie^{i\phi} \sin\frac{\theta}{2} \right) |S, \uparrow\rangle_y + \left(\cos\frac{\theta}{2} + ie^{i\phi} \sin\frac{\theta}{2} \right) |S, \downarrow\rangle_y \right] \right), \end{aligned} \tag{S2.6}$$

where C_{L,m_l,S,m_s} is a Clebsch-Gordan coefficient. Defining $q_\pm$ as

$$q_+ := \langle a|\hat{R}_{+1}| \sum C_{L,m_l,S,m_s} |L, m_l\rangle_b |S, \uparrow\rangle_y \tag{S2.7}$$

$$= \langle a|\hat{R}_{-1}| \sum C_{L,m_l,S,m_s} |L, m_l\rangle_b |S, \downarrow\rangle_y , \tag{S2.8}$$

$$q_- := \langle a|\hat{R}_{+1}| \sum C_{L,m_l,S,m_s} |L, m_l\rangle_b |S, \downarrow\rangle_y \tag{S2.9}$$

$$= \langle a|\hat{R}_{-1}| \sum C_{L,m_l,S,m_s} |L, m_l\rangle_b |S, \uparrow\rangle_y , \tag{S2.10}$$

then, for our XFMR measurement, $F^1_{\pm 1}$ can be expressed using $q_\pm$ as below;

$$F^1_{+1} = \sum_b \frac{1}{2} \frac{p(b)}{E_b - E_a + E_p + i\Gamma/2} \left| \left(\cos\frac{\theta}{2} - ie^{i\phi} \sin\frac{\theta}{2} \right) q_+ + \left(\cos\frac{\theta}{2} + ie^{i\phi} \sin\frac{\theta}{2} \right) q_- \right|^2 , \tag{S2.11}$$

$$F^1_{-1} = \sum_b \frac{1}{2} \frac{p(b)}{E_b - E_a + E_p + i\Gamma/2} \left| \left(\cos\frac{\theta}{2} - ie^{i\phi} \sin\frac{\theta}{2} \right) q_- + \left(\cos\frac{\theta}{2} + ie^{i\phi} \sin\frac{\theta}{2} \right) q_+ \right|^2 . \tag{S2.12}$$

For the cases that spin is perpendicular to the X-ray direction, i.e., $\theta = 0$, or $\theta = \pi/2$ and $\phi = 0$, Eqs. (S2.11) and (S2.12) lead to $F^1_{+1} = F^1_{-1}$, consequently XFMR signals vanish ($\Delta\gamma = 0$).

For $\phi = \frac{\pi}{2}$, one can calculate as below;

$$F^1_{-1} - F^1_{+1} = \sum_b \frac{p(b)}{E_b - E_a + E_p + i\Gamma/2} \sin\theta (|q_-|^2 - |q_+|^2). \tag{S2.13}$$

Hence, XFMR signals at $\phi = \frac{\pi}{2}$ are given by

$$\Delta\gamma^{\mathrm{XFMR}} = \sin\theta \Delta\gamma^{\mathrm{XMCD}}, \tag{S2.14}$$

where

$$\Delta\gamma^{\mathrm{XMCD}} := C\Im \left[\sum_b \frac{p(b)}{E_b - E_a + E_p + i\Gamma/2} (|q_-|^2 - |q_+|^2) \right] . \tag{S2.15}$$

Eq. (S2.15) gives usual XMCD signals when the spin moment points to the y-direction. Thus, according to Eq. (S2.14), XFMR spectra are expressed by projected components of XMCD spectra along the X-ray beam direction.

S3. XMCD Measurements

Figures S2 (a) and (b) present X-ray absorption spectroscopy (XAS) and XMCD data around the Ni $L_{2,3}$ edges for the Ta(2)[Pt(2)/Py(5)]$_6$ and the Pt(10)/Py(30) samples. XAS data were obtained in the total-electron-yield (TEY) mode. An external magnetic field was applied perpendicular to the sample surface. I_+ and I_- represent the XAS data obtained using right and left circular polarized X-rays, divided by incident (I0) X-ray intensity. XMCD data were acquired by subtracting these spectra.

Figure S3 shows the angular dependence ($\theta = 0°, 70°$) of XMCD data for the Pt(10)/Py(30) sample. θ is defined as an angular between the direction of the incident X-ray and the surface normal of the sample. All spectra are normalized by the intensities at the L_2 edge. The intensity at the L_3 edge is decreased at

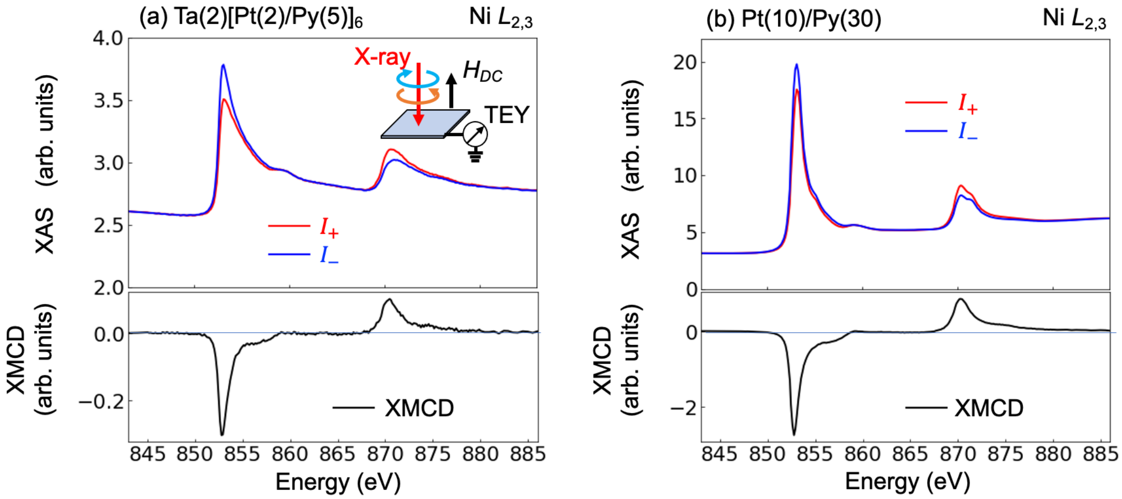

Fig. S2: (a)(b) XAS and XMCD spectra around the Ni $L_{2,3}$ edges of (a) Ta(2)[Pt(2)/Py(5)]$_6$ and (b) Pt(10)/Py(30). I_+ and I_- represent the data obtained using right and left circular polarized X-rays, respectively. Inset in the figure (a) illustrates experimental setup of XMCD measurements.

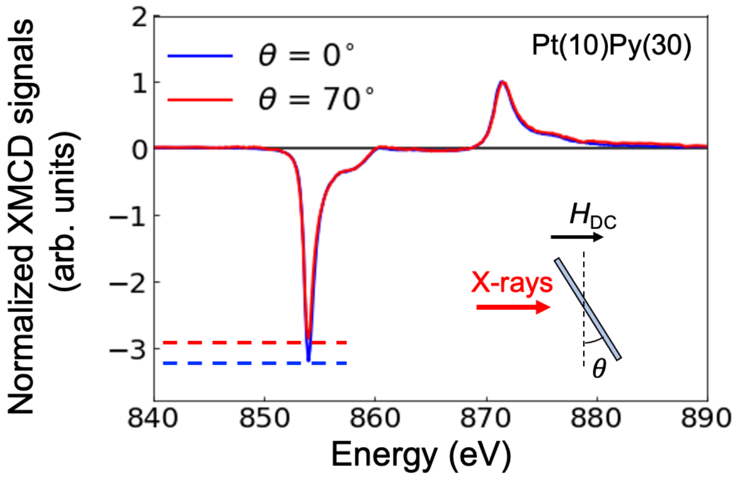

Fig. S3: Angular (θ) dependence of XMCD signals of the Pt(10)/Py(30). The inset shows the definition of θ These spectra are normalized by the intensities at the L_2 edge. Dashed lines present the intensities at the L_3 edge. All data were acquired by the TEY method.

$\theta = 70°$. The ratios of orbital magnetic moments to spin magnetic moments ($r = \mathbf{m}_L/\mathbf{m}_S$) are acquired using the Eq. (4) in the main text: $r = 0.12$ at $\theta = 0°$ and $r = 0.08$ at $\theta = 70°$. This fact indicates that the $\mathbf{m}_L$ prefers to point in the direction of the surface normal of the sample.

It is expected that the XMCD signals show no significant dependence on the in-plane direction because the samples and SiN membranes are both polycrystallines.

S4. Bayesian Analysis for XFMR data

Defining $\boldsymbol{D} = \{E_i, I_i^{\mathrm{XFMR}}\}$ as a obtained dataset, where E_i and I_i^{XFMR} represent incident X-ray energy and XFMR intensity data, and $\boldsymbol{\theta}$ as a parameter set, Bayes' theorem between $\boldsymbol{D}$ and $\boldsymbol{\theta}$ is expressed by below equation [3, 4, 5]:

$$p(\boldsymbol{\theta}|\boldsymbol{D}, b) = p(\boldsymbol{D}|\boldsymbol{\theta}, b)\frac{p(\boldsymbol{\theta})}{p(\boldsymbol{D}, b)}, \tag{S4.1}$$

where $p(\boldsymbol{\theta}|\boldsymbol{D}, b)$ is a posterior probability distribution, and $p(\boldsymbol{D}|\boldsymbol{\theta}, b)$ is a conditional probability of $\boldsymbol{D}$ under the condition of $\boldsymbol{\theta}$, and $p(\boldsymbol{\theta})$ is a prior probability for θ. b is a quasi-inverse temperature defined as $b := 1/\sigma^2$, where σ is the standard deviation of the noises ϵ in the dataset. Defining a model function as $f(E_i; \boldsymbol{\theta})$, I_i^{XFMR} can be written as

$$I_i^{\mathrm{XFMR}} = f(E_i; \boldsymbol{\theta}) + \epsilon_i. \tag{S4.2}$$

Assuming parameters of the noises in the dataset are distributed in a normal distribution, $p(\boldsymbol{D}|\boldsymbol{\theta}, b)$ is given by

$$p(\boldsymbol{D}|\boldsymbol{\theta}, b) = \left(\frac{b}{2\pi}\right)^{N/2} \exp(-Nb\mathcal{E}(\boldsymbol{\theta})), \tag{S4.3}$$

where N is the number of the data, and $\mathcal{E}(\boldsymbol{\theta})$ is an error function, which is defined as

$$\mathcal{E}(\boldsymbol{\theta}) := \frac{1}{2N} \sum_i (I_i^{\mathrm{XFMR}} - f(E_i; \boldsymbol{\theta}))^2. \tag{S4.4}$$

The noise distribution b can be estimated by the below equation

$$\hat{b} = \arg\min_b \mathcal{F}(b) \tag{S4.5}$$

where,

$$\mathcal{F}(b) := -ln\mathcal{Z}(b) \tag{S4.6}$$

with

$$\mathcal{Z}(b) := p(\boldsymbol{D}, b) = \int p(\boldsymbol{D}|\boldsymbol{\theta}, b)p(\boldsymbol{\theta})d\boldsymbol{\theta}. \tag{S4.7}$$

Thus, the posterior probability distribution $p(\boldsymbol{\theta}|\boldsymbol{D}, b)$ can be expressed with the estimated quasi-inverse temperature $\hat{b}$ as

$$p(\boldsymbol{\theta}|\boldsymbol{D}, \hat{b}) = \left(\frac{\hat{b}}{2\pi}\right)^{N/2} \exp(-N\hat{b}\mathcal{E}(\boldsymbol{\theta}))\frac{p(\boldsymbol{\theta})}{\mathcal{Z}(\hat{b})} \propto \left(\frac{\hat{b}}{2\pi}\right)^{N/2} \exp(-N\hat{b}\mathcal{E}(\boldsymbol{\theta}))p(\boldsymbol{\theta}). \tag{S4.8}$$

In the analysis for the XFMR data, we assumed that the shapes of XFMR spectra around the Ni L_3 and L_2 edges should be almost the same as those of static XMCD, specifically a model function $f(E_i; \boldsymbol{\theta})$ utilized for the Bayesian analysis of the data around each edge was deemed to be a constant multiple of that of the static XMCD spectrum. Thus we prepared the parameter sets of $\boldsymbol{\theta} = \{C_1, C_2\}$, where C_1 and C_2 represent the constant coefficients of multiplication for the spectra around the Ni L_3 and L_2 edges, respectively, i.e.,

$$f(E_i; \boldsymbol{\theta}) = C_1\mathcal{L}(E_i)_{L_3}^{\mathrm{XMCD}} + C_2\mathcal{L}(E_i)_{L_2}^{\mathrm{XMCD}}, \tag{S4.9}$$

where $\mathcal{L}(E_i)_{L_{2(3)}}^{\mathrm{XMCD}}$ represent the data of static XMCD spectra around the Ni $L_{2(3)}$ edges. Replica exchange Monte Carlo method was employed to calculate Eq. (S4.8) by sampling the parameter $\boldsymbol{\theta}$. Subsequently, the posterior probability distribution of r $[p(r|\boldsymbol{D}, \hat{b})]$ was acquired from $p(\boldsymbol{\theta}|\boldsymbol{D}, \hat{b})$ through utilization of Eq. (4) in the main text. Furthermore the estimated XFMR spectra, as shown by red and blue solid lines in Figs. 2 (a) and (b) in the main text, were obtained using the maximum values of $p(C_1|\boldsymbol{D}, b)$ and $p(C_2|\boldsymbol{D}, b)$.

References

[1] K. Carva, D. Legut and P. M. Oppeneer, Influence of laser-excited electron distributions on the X-ray magnetic circular dichroism spectra: Implications for femtosecond demagnetization in Ni, EPL., 86, 57002 (2009).

[2] Lovesey, S. W. and Collins, S. P., X-ray Scattering and Absorption by Magnetic Materials, Oxford Series on Synchrotron Radiation, No. 1 (1996).

[3] Thomas, B., An essay towards solving a problem in the doctrine of chances, Phil. Trans. Toy. Soc. 53, 370 (1763).

[4] Nagata, K., Sugita, S. and Okada, M., Bayesian spectral deconvolution with the exchange monte carlo method., Neural Networks 28, 82-89 (2012).

[5] Tokuda S., Nagata. K, Okada M., Simultaneous Estimation of Noise Variance and Number of Peaks in Bayesian Spectral Deconvolution, J. Phys. Soc. Jpn., 86, 024001 (2017).
